# A spray-as-you-go airway topical anesthesia attenuates cardiovascular responses for double-lumen tube tracheal intubation

**DOI:** 10.1186/s12871-022-01749-8

**Published:** 2022-07-02

**Authors:** Changlin Chen, Di Wen, Yizheng Wang, Hongqiong Li, Qi Yu, Mao Li

**Affiliations:** Department of Anesthesiology, Affiliated Hospital of North Sichuan Medical College, Nanchong, 637000 China

**Keywords:** Spray-as-you-go, Superior laryngeal nerve block, Transtracheal injection, Airway topical anesthesia, Double-lumen tube, Intubation

## Abstract

**Background:**

Spray-as-you-go (SAYGo) airway topical anesthesia and nerve block are common techniques used during awake tracheal intubation. However, their effects have not been described during double-lumen tube intubation. We report on a prospective randomized study that aimed to compare the intubation effects of SAYGo and nerve block patients undergoing thoracic surgery.

**Methods:**

Sixty-six American Society of Anesthesiologists (ASA) physical status I and II patients were scheduled to undergo double-lumen tube (DLT) tracheal intubation for thoracic surgery. The patients were randomly assigned into control (Group C), ultrasound (Group U), and flexible intubation scope (Group F) groups with 22 cases in each group. Patients in Group C were induced with a standard anesthetic regimen. Patients in Groups U and F were treated with superior laryngeal nerve (SLN) block combined with transtracheal injection (TTI) and given a SAYGo airway topical anesthesia before intubation. Hemodynamic variables during intubation process were recorded as the primary outcome. Additional patient data were recorded including the occurrence of adverse events, the level of hoarseness, the occurrence of sore throats, memory function and the level of patient satisfaction with anesthesia.

**Results:**

The blood pressure (BP) and heart rate (HR) of patients in group C was significantly increased 1 min after tracheal intubation (*P* < 0.05) compared to before anesthesia. The BP and HR of patients in Groups U and F remained stable. 10 cases of hypertension were observed in Group C, 6 cases in Group U and 1 case in Group F. In Group C, tachycardia was observed in 9 patients along with 9 cases in Group U and 4 cases in Group F. In Group U, 4 patients experienced puncture and bleeding were and 8 patients had a poor memory of TTI. No significant differences were found in the incidence of hoarseness, sore throats, and satisfaction with anesthesia in postoperative follow-up.

**Conclusions:**

SAYGo airway topical anesthesia and SLN block combined with the TTI technique can inhibit the cardiovascular response during DLT tracheal intubation. The SAYGo technique has fewer complications and more advantages compared to other approaches.

## Background

In thoracic surgery, general anesthesia with DLT tracheal intubation is commonly used to achieve single-lung ventilation that is required during the operation [1]. Due to the large diameter, length, and hard texture of the DLT, strong stimulation of the tube induced fluctuations in hemodynamics can increase the incidence of cardiovascular adverse events [2, 3]. Approaches to reduce stress during DLT tracheal intubation have been a focus of clinical research. Vasoactive drugs and anesthetics are commonly used to inhibit the stress response during intubation [4] yet the optimum dosing of these drugs remains challenging in the clinic and can cause significant hemodynamic fluctuations [5, 6].

The strong response of DLT tracheal intubation is caused by the direct mechanical stimulation by the laryngoscope on the larynx and trachea and catheter during the intubation process [7]. During intubation, stress can be reduced by applying topical anesthesia to the airway to align with the concept of precise anesthesia and rapid recovery (enhanced recovery after surgery, ERAS) [8]. The base of the tongue, epiglottis, piriform fossa, and vallecula are all innervated by the SLN [9]. Therefore, SLN block combined with transtracheal topical anesthesia can effectively inhibit the strong stress response in the throat and trachea caused by tracheal intubation. The anesthetic effect of this method has been demonstrated during conscious intubation [10]. Ultrasound-guided nerve block and TTI increase the success rate of nerve block and puncture and improve the reliability of the anesthesia [11, 12].

Flexible intubation scope (FIS) can also be used as it is less traumatic and offers high visibility. Spraying topical anesthetics through the working channel of the FIS using a SAYGo method can gradually complete the airway mucosal surface anesthesia from the throat to the bronchus [13]. As this procedure does not require other equipment or complex anatomical knowledge, it can be used to effectively reduce trauma and is commonly used in operations involving awake intubation and bronchoscopy [14, 15].

The above two airway topical anesthesia methods have been widely used in awake intubation and have demonstrated clinical efficacy yet there is a lack of studies involving DLT intubation. We designed a prospective randomized study to compare the application effects of two airway topical anesthesia methods in DLT intubation. We hypothesized that the SAYGo topical anesthesia can attenuate cardiovascular responses for DLT intubation and is advantageous compared to other approaches.

## Materials and methods

This randomized, double-blinded, prospective clinical trial was registered with the Chinese registry of clinical trials at http://www.chictr.org.cn (ChiCTR2100042847; 30/01/2021). The study was approved by the Ethics Committee of the Affiliated Hospital of North Sichuan Medical College and all patients were recruited under written informed consent. Sixty-six patients with ASA I-II physical status thoracic surgery who were between 18–65 years old and required DLT tracheal intubation were included in the study. A random number table created by statisticians using SPSS statistical software was used to randomly assign patients to the study groups control (Group C), ultrasound (Group U), and flexible intubation scope (Group F) groups with 22 patients in each group. The exclusion criteria for the study were patients with anticipated difficult airway, allergies to topical anesthesia or other anesthetics, trauma or infection at the puncture site, coagulopathy, pregnant women, and individuals with communication difficulties.

All patients were injected with penehyclidine hydrochloride (0.5 mg intramuscular) 30 min before the operation. NIBP, HR, electrocardiography, pulse oximetry, end-tidal carbon dioxide, and bispectral index (BIS) were initiated in the operating theatre. A peripheral venous channel was established. After intravenous injection of midazolam (0.03 mg/kg) and sufentanil (0.1 ug/kg), a radial artery puncture was performed to monitor the direct arterial pressure. Before the start of anesthesia 5 ml/kg of Ringer's lactate was intravenously infused.

Patients in Group C were induced by an intravenous bolus injection of sufentanil (0.3 ug/kg), etomidate (0.3 mg/kg), and rocuronium (0.8 mg/kg). Direct laryngoscopy and DLT tracheal intubation were performed when the mask was ventilated with pure oxygen for 3 min and the BIS value was < 60. After successful intubation, the same operator used a FIS to complete the adjustment and positioning of the dual-cavity position. After 5 min, the patient was changed from the supine to the lateral position.

**Fig. 1:**
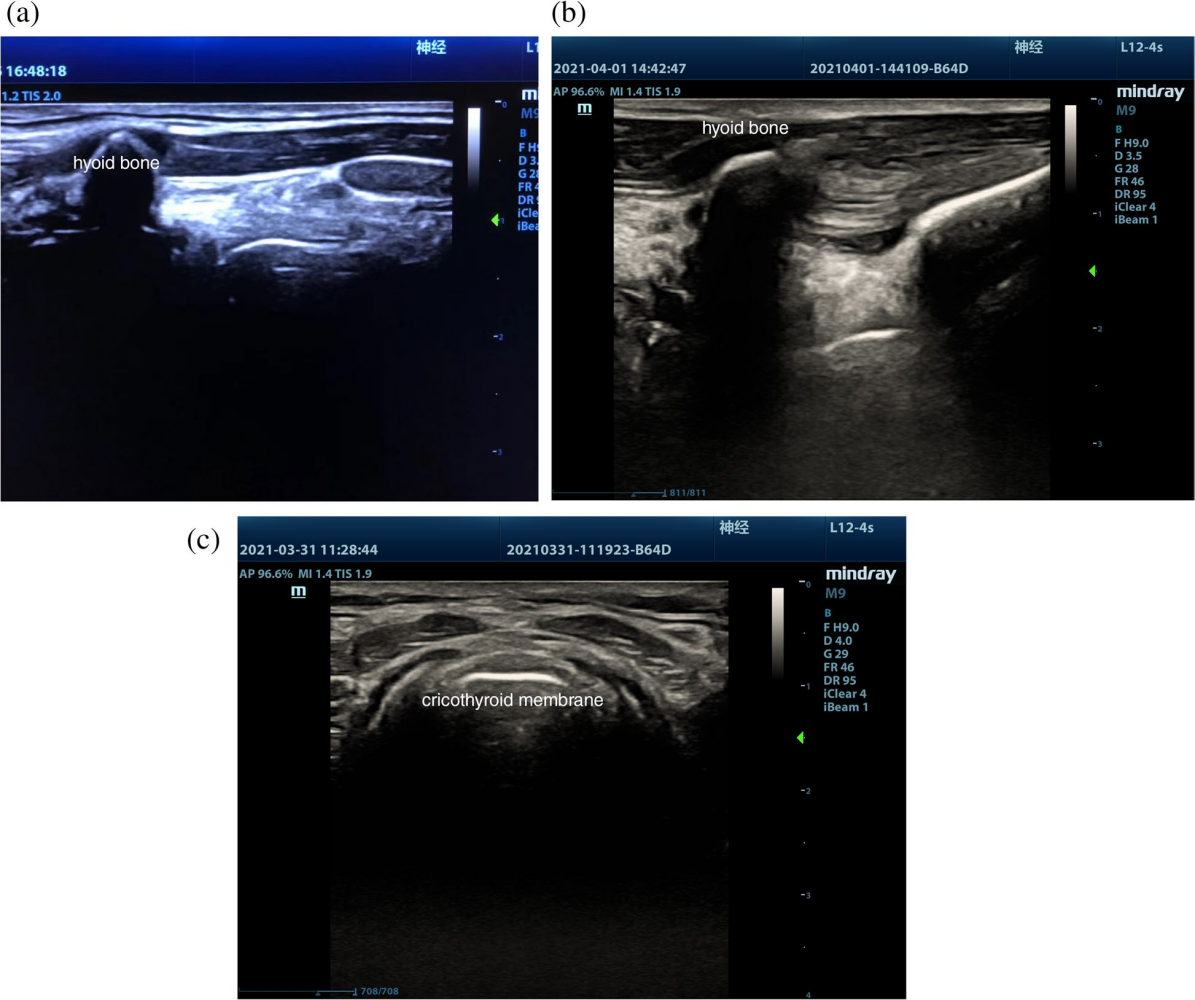
Ultrasound-guided SLN block and transtracheal injection. Note: **a** The tissue structure around the SLN under sagittal ultrasound, **b** the sagittal out-of-plane puncture under ultrasound. 2 ml of 2% lidocaine solution was injected into the thyroglossal membrane. **c** The location of the cricothyroid membrane under coronal ultrasound

Patients in Group U received bilateral SLN block and TTI topical anesthesia [16, 17]. A high-frequency (13.6 MHz, 6 cm) linear array probe was used with the hyoid bone as the initial positioning mark. The probe was then moved downwards and outwards. The thyrohyoid periosteum is located between the hyoid bone and the thyroid cartilage. The position of the SLN was determined by rotating the probe to the sagittal position (Fig. 1a). A 22 G puncture needle was used to inject 2 ml of 2% lidocaine using an out-of-plane method (Fig. 1b). The cricothyroid puncture was marked by the thyroid cartilage and the probe was translated downwards. The high-bright line echo between the thyroid cartilage and the cricoid cartilage was the cricothyroid (Fig. 1c). After a successful puncture using the in-plane technique, 3 ml of 2% lidocaine was injected. Venous induction intubation was performed 5 min later and the same method was used for patients in Group C.

**Fig. 2:**
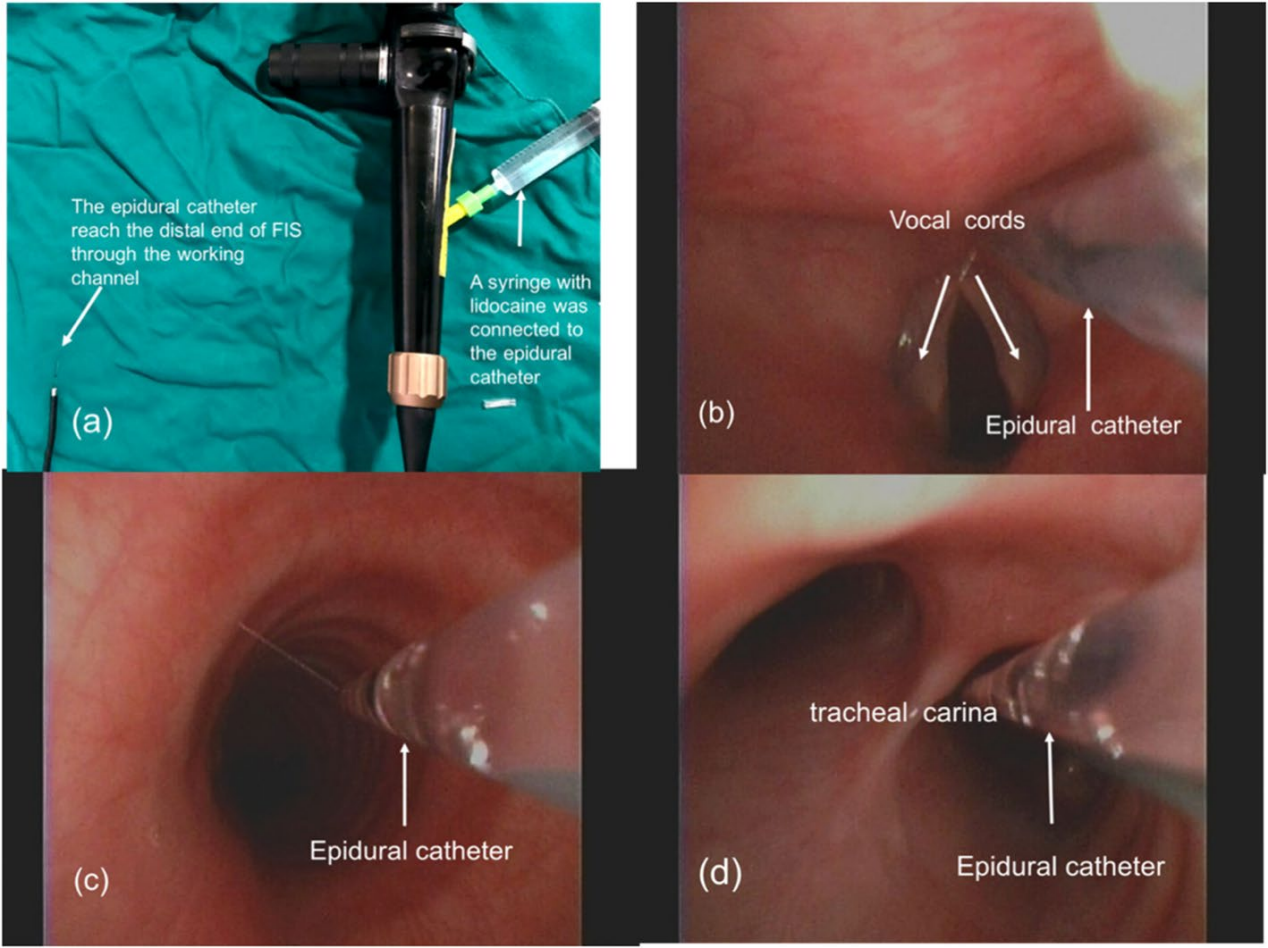
The technique of spray-as-you-go airway topical anesthesia. Note: **a** A FIS with an epidural catheter was inserted into the working channel. One end of the catheter was connected to a syringe containing 2% lidocaine, **b** the spray on the glottis under the FIS, **c** The anesthetic was sprayed through the side hole of the epidural catheter, **d** Spraying of the carina and bronchi under the FIS

Patients in Group F were intravenously injected with the same anesthetics as the patients in group C. SAYGo technology was used as a topical anesthesia in the airways after 3 min of pure oxygen ventilation with a mask. An assistant placed the dental pads and lifted the lower jaw. The operator used a FIS equipped with an epidural catheter in the working channel (Fig. 2a) to inject 1 ml of 2% lidocaine into the bilateral piriform crypts, epiglottis, and glottis under the direct view of the mouth. The endoscope was slowly positioned in the subglottic trachea to the main bronchus, and advanced in the trachea whilst continuously spraying 2% lidocaine totaling 3 ml (Fig. 2b-d). In cases were the SpO_2_ was lower than 90% during epithelial anesthesia, the operation was suspended and tracheal intubation was performed after the epithelial anesthesia was completed with mask ventilation for 3 min. The tracheal intubation was the same as those used in patients in Group C.

The intubation and positioning were performed by an anesthesiologist who was skilled in the operation and was blinded to the experimental groups. In cases were the intubation and positioning were unsuccessful on the first attempt, the patient was withdrawn from the study. During the anesthesia process, an independent person blinded to the experimental protocol and grouping was responsible for observing and recording the experimental data. The primary outcomes were blood pressure and heart rate before anesthesia (T0), before intubation (T1), and at 1 (T2), 3 (T3) and 5 min (T4) after intubation, and immediately after the position change (T5). The secondary outcomes included the occurrence of adverse events, patients with hoarseness, sore throats, and the level of satisfaction with anesthesia were followed for 24 h after surgery.

During the observations, if hypertension (blood pressure > 30% of the base value or SBP > 160 mmHg) or hypotension (blood pressure < 30% of the base value or SBP < 90 mmHg), Nicardipine (0.2 mg) or Phenylephrine (50 ug) was given by intravenous injection. If tachycardia (HR > 100 beats/min) or bradycardia (HR < 50 beats/min) occurred, Esmolol (20 mg) or Atropine (0.5 mg) was given intravenously and repeated administration if necessary.

### Statistical analysis

The sample size was calculated according to the preliminary experimental data and based on the fact that the blood pressure 1 min after intubation was significantly higher compared to before anesthesia. A 20% reduction in blood pressure was considered effective. The test level was a = 0.05 and the test efficiency was (1-b) = 0.8. The distribution ratio of the three patient groups was 1:1:1. PASS 15.0 software was used to calculate the sample content for 17 cases in each group. Considering that 20% of the patients were withdrawn, 22 cases were included in each group, with a total sample size of 66 cases.

The data were analyzed using SPSS 23.0 statistical software. The continuous variables are expressed as the mean ± standard deviation and the categorical variables are expressed as proportions (%). The Shapiro–Wilk test was used to test the distribution of the data. Normally distributed data were analyzed using an analysis of variance. Repeated measures data were analyzed using a repeated-measures analysis of variance. The categorical data were analyzed using a Chi-square test or Fisher's exact test. *P*-values of < 0.05 were considered statistically significant.

## Results

A total of 66 patients were included in this study. In Group C, 3 patients were withdrawn from the study due to intubation or positioning on the first attempt along with 1 patient in Group U and 1 patient in Group F. Finally, 61 patients were included in the statistical analysis. No significant differences were detected between the three groups of patients in age, gender, weight, height, body mass index, ASA status, intubation position, and catheterization time. Also, no significant differences in baseline vital signs were detected across all of the patients at admission (Table 1).

**Table 1:** Summary of the baseline characteristics of the study participants

Basic information	Group C (*n* = 19)	Group U (*n* = 21)	Group F (*n* = 21)
Age (year)	54.89 ± 7.48	49.38 ± 11.56	52.24 ± 11.67
Male/female	11/8	12/9	13/8
Height (cm)	160.26 ± 6.00	163.57 ± 6.51	164.43 ± 8.54
Weight (kg)	60.89 ± 9.76	58.90 ± 9.58	62.24 ± 10.13
BMI	23.65 ± 3.15	21.95 ± 2.77	22.95 ± 2.77
ASA classification (I/II)	4/15	6/15	3/18
Intubation site (left/right)	13/6	14/7	13/8
With tube time (min)	168.63 ± 56.92	197.14 ± 74.31	176.05 ± 50.08

**Fig. 3:**
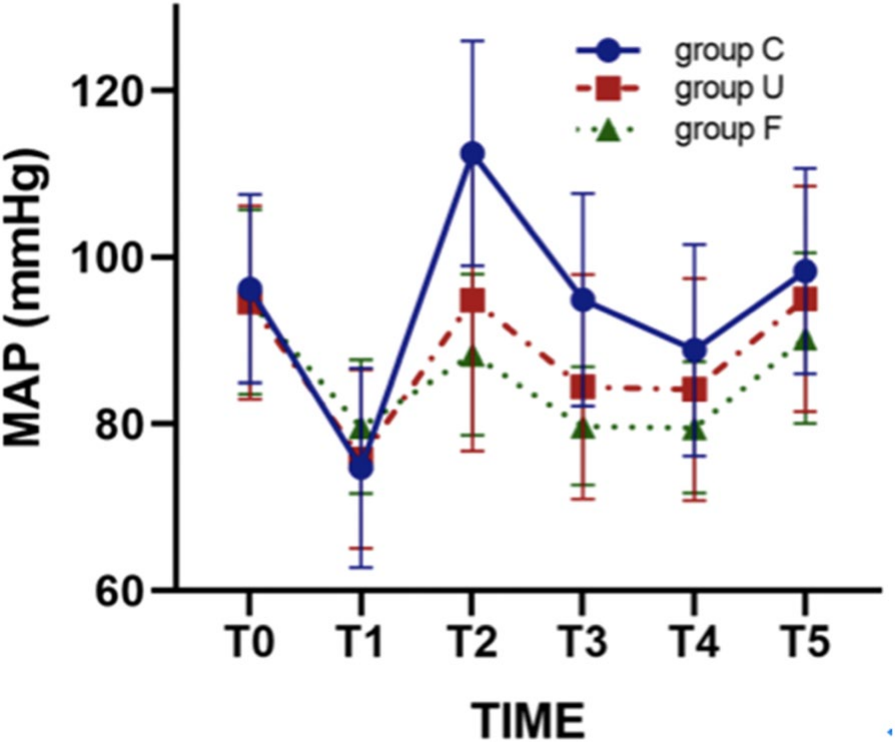
The observed trends in mean arterial pressure (MAP) of the three groups of patients

**Fig. 4:**
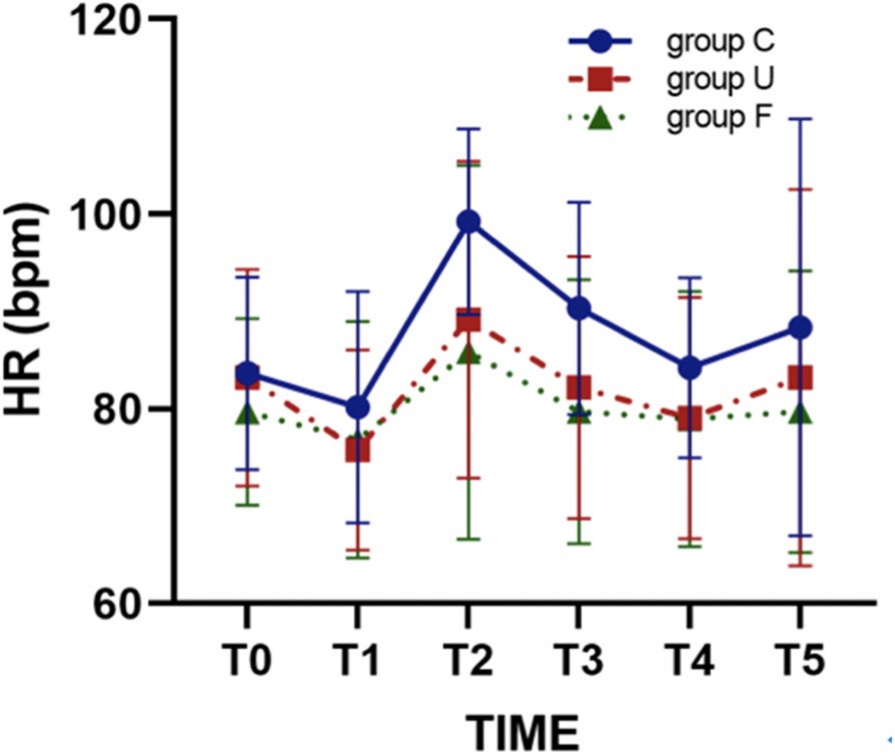
The observed trends in heart rate (HR) of the three groups of patients

The BP of patients in the three groups was significantly lower before intubation compared to before anesthesia (*P* < 0.05). The BP of patients in Group C increased significantly after tracheal intubation (*P* < 0.05) whilst the BP of patients in Groups U and F remained stable (Table 2, Fig. 3). Compared to before anesthesia, the HR of patients in Group C increased significantly at 1 and 3 min after tracheal intubation (*P* < 0.05) whilst the HR of patients in Groups U and F did not significantly fluctuate (Table 3, Fig. 4). The operation times of the topical anesthesia in patients in Group U were 116.8 ± 10.1 s longer compared to Group F at 93.0 ± 10.0 s (*P* < 0.05). The incidence of hypertension in patients in Group C was significantly higher in 10 cases (52.6%) compared to patients in Group F (4.8%) (*P* < 0.05) and Group U in which 6 cases (28.6%) were observed. There were 9 cases of tachycardia in Group C, 9 cases in Group U, 4 cases in Group F. In Group U, 4 patients had throat bleeding when the glottis was exposed by the laryngoscope, and 8 patients had poor memory related to the operation during the postoperative follow-up. The patients in Group F did not have hypoxemia during the operation and had no bad memories after the operation (Table 4). No significant differences were observed in postoperative hoarseness, the incidence of sore throats, and satisfaction with anesthesia between the three groups (Table 5).

**Table 2:** Summary of the changes in mean arterial pressure (MAP)

	Point in time	Group C (*n* = 19)	Group U (*n* = 21)	Group F (*n* = 21)
MAP (mmHg)	T0	96.21 ± 11.26	94.57 ± 11.58	94.62 ± 11.04
	T1	74.74 ± 11.92^*^	75.81 ± 10.69^*^	79.67 ± 7.97^*^
	T2	112.47 ± 13.48^*^	94.81 ± 18.02	88.33 ± 9.65
	T3	94.89 ± 12.73	84.48 ± 13.45^*^	79.76 ± 7.09^*^
	T4	88.84 ± 12.65^*^	84.14 ± 13.26^*^	79.57 ± 7.84^*^
	T5	98.32 ± 12.33	95.00 ± 13.52	90.29 ± 10.22

^*^P < 0.05 Compared to the base value

**Table 3:** Summary of the changes in HR

	Point in time	Group C (*n* = 19)	Group U (*n* = 21)	Group F (*n* = 21)
HR (bpm)	T0	83.63 ± 9.87	83.19 ± 11.12	79.67 ± 9.60
	T1	80.16 ± 11.91	75.76 ± 10.29^*^	76.81 ± 12.15
	T2	99.21 ± 9.55^*^	89.14 ± 16.26	85.81 ± 19.20
	T3	90.32 ± 10.90^*^	82.19 ± 13.46	79.71 ± 13.55
	T4	84.21 ± 9.25	79.05 ± 12.40	78.95 ± 13.12
	T5	88.37 ± 21.40	83.19 ± 19.33	79.71 ± 14.46

^*^P < 0.05 Compared to the base value

**Table 4:** Summary of the occurrence of adverse events

Adverse events	Group C (*n* = 19)	Group U (*n* = 21)	Group F (*n* = 21)
High blood pressure	10	6	1^†^
Tachycardia	9	9	4
Low blood pressure	0	3	0
Bradycardia	0	0	0
Throat bleeding	0	4	0

^†^*P* < 0.05 Compared to group C

**Table 5:** Summary of postoperative follow-up

	Group C (*n* = 19)	Group U (*n* = 21)	Group F (*n* = 21)
Hoarse (mild/medium/heavy)	6/3/0	7/3/0	8/3/0
Sore throat (mild/medium/severe)	0/2/0	0/1/0	2/0/0
Patient satisfaction score	94.47 ± 3.42	95.19 ± 3.78	96.76 ± 2.72

## Discussion

This study found that the BP and HR of patients in group C increased sharply 1 min after intubation and returned to baseline within 3 to 5 min. The observations agreed with a previous study which showed that the maximal increases in BP and HR occurred 1 to 2 min after DLT intubation in the control group and returned to baseline within 5 min [18]. Byung-Hee et al. used 3 ug/kg sufentanil for DLT intubation and found that HR was significantly increased after intubation whilst BP was not significantly increased potentially related to the combined use of propofol [19]. Whilst we found the hemodynamics in group U and F remained stable after intubation, both SLN block combined with TTI and SAYGo airway topical anesthesia effectively attenuated cardiovascular responses to DLT tracheal intubation. Compared to the group F, there was no advantage in group U. The findings may be due to the need for ultrasound equipment, the risk of bleeding risks and corresponding contraindications for the invasive operation during nerve block and TTI in group U.

SLN block can effectively inhibit the sensory nerves of the throat and tongue mucosa. This block effectively reduces the adverse irritation caused by the laryngoscope during intubation and the strong stimulation of the DLT in the throat, which has been reported in awake DLT intubation [20]. TTI is a classic method of intratracheal mucosal anesthesia and is also an effective technique commonly used for conscious intubation [21]. Michael et al. showed that TTI can meet the requirements of intubation with stable cardiopulmonary indices immediately after insertion of the ETT [22] which are similar to our data.

One of the key points in SLN block and TTI is accurate positioning. According to previous studies, the success rate of the empirical blind method in locating the cricothyroid membrane is less than 50% and even lower for obese patients. In contrast, the accuracy of ultrasound-assisted positioning can be increased to 100% [23–25]. Ultrasound positioning was used to guide the puncture needle in SLN block and the cricothyroid membrane puncture. All the patients experienced abnormal sensations and a cough response after SLN block and TTI. This study confirmed that this method can be used to reduce the stress response of DLT tracheal intubation and stabilize the hemodynamic effects in patients during intubation.

Patients in group F showed stable hemodynamics after intubation and had the lowest incidence of hypertension and tachycardia amongst the three groups. Previous studies have found that the SAYGo technique with a epidural catheter provides adequate airway anesthesia and stable hemodynamics for awake fiber-optic intubation(AFOI) [26, 27]. However, BP and HR have been shown to improve rapidly to basal values after intubation using the SAYGo topical anesthesia [28]. Under the synergistic effect of anesthetics and SAYGo topical anesthesia, the hemodynamics of patients in group F were stable after intubation.

Flexible bronchoscopy is the gold standard for guiding awake tracheal intubation and is an effective method for airway topical anesthesia [21, 29]. Also, bronchoscopy is often used to judge and adjust the position of the intubation in DLT tracheal intubation [30]. In the awake state, the SAYGo technique usually requires appropriate sedation and analgesia that is gradually completed with the cooperation of the patient and can be lengthy [31]. However, the SAYGo technique was performed after induction of anesthesia in our study and so no patients experienced coughing and gagging that often occurs during AFOI [27, 32]. Also, the operation was completed within 2 min and so all patients did not develop hypoxemia during topical anesthesia. However, during the operation, an assistant is often required to hold the lower jaw, fix a dental pad, and complete the drug injection.

In this study, the purpose of spraying the topical anesthetic solution using an epidural catheter in the working channel of the FIS was to ensure that the drug solution was more evenly distributed on the surface of the airway mucosa. The epidural catheter technique has been described in multiple studies and shown adequate topical anesthesia for awake intubation [26, 27].

In our study, we found the number of cases of hypertension and tachycardia in the group F were lower than those in the group U. Thomas et al. [27] evaluated the application of local anesthesia in the lower airway via a SAYGo technique and found that it was as effective of transtracheal block. However, the DLT in our study was longer than the conventional tube. The operation of DLT tracheal intubation directly stimulated the entire trachea including the bronchus. The local anesthetic in group F was sprayed directly from the mouth to the bronchus under direct vision. However, the distribution of local anesthetic in the carina and bronchi in group U may be affected by the cough response of the patient. The method of increasing the sample size and using topical anesthesia to develop colors will help to further validate these hypotheses.

In group U, 4 patients had bloodstains on the glottis when it was exposed by the laryngoscopy and 8 patients had poor memory after surgery. In a randomized controlled trial [28], it was reported that 47% of patients who underwent TTI had poor memory after surgery, while only 7% of patients who underwent the SAYGo technique. Coughing caused by TTI can lead to the risk of accidental injection of topical anesthetics into large blood vessels, topical anesthetic poisoning, bleeding, and airway damage. Also, severe coughing has the associated risk of reflux and aspiration [33, 34].

Lidocaine is an effective topical anesthetic that can be used safely at a dose of 9 mg/kg in airway mucosal anesthesia [35]. In this study, each patient undergoing local anesthesia received 100 mg of lidocaine. There were no significant differences in the incidence of postoperative sore throat, hoarseness, and the satisfaction score of anesthesia between the three groups. A recent meta-analysis of lidocaine for postoperative sore throat indicated that intracuff and intravenous lidocaine are effective in minimizing the risk of postoperative sore throat, but lidocaine jelly and spray are not [36].

This study had several limitations including the lack of monitoring of blood catecholamine levels. The BP of the two local anesthesia groups of patients, especially the group F, was lower than the baseline values after anesthesia induction and intubation. This may be due to the use of conventional intravenous anesthesia induction drugs. Effective airway topical anesthesia can maintain circulatory stability during the induction intubation process and may reduce anesthetic dose. The optimal anesthesia induction medication regimen combined with the topical anesthesia technique in the process of DLT tracheal intubation requires further investigation.

In summary, the use of topical airway anesthesia during DLT tracheal intubation can effectively inhibit adverse cardiovascular reactions and stabilize hemodynamics during intubation. As a common auxiliary device in the process of DLT tracheal intubation, bronchoscopy can be used to adjust and locate the position of the intubation, and can be combined with the SAYGo airway mucosal anesthesia method of the bronchoscopy working channel.

## Data Availability

Data have been uploaded successfully to the Chinese registry of clinical trials at https://www.chictr.org.cn/historyversionpub.aspx?regno=ChiCTR2100042847 The datasets used and/or analyzed during the current study are available from the corresponding author upon reasonable request.
